# HUMANIN produced by human efferocytic macrophages promotes the resolution of inflammation

**DOI:** 10.1038/s41419-025-07909-1

**Published:** 2025-08-28

**Authors:** Mélissa Maraux, Mathieu Vetter, Ludivine Dal Zuffo, Francis Bonnefoy, Audrey Wetzel, Alexis Varin, Baptiste Lamarthée, Olivier Tassy, Didier Ducloux, Philippe Saas, Thomas Cherrier

**Affiliations:** 1 Université Marie et Louis Pasteur, EFS, INSERM, UMR RIGHT, F-25000 Besançon, France, LabEx LipSTIC, Besançon, France; 2 Institut de Génétique et de Biologie Moléculaire et Cellulaire (IGBMC), Inserm U1258, Université de Strasbourg, Illkirch Graffenstaden, France; 3 CHRU Besançon, Service de Néphrologie, Besançon, France; 4 Université Grenoble Alpes, INSERM U1209, CNRS UMR 5309, EFS AuRA, Institut pour l’Avancée des Biosciences (IAB), DIMAC, GRENOBLE, France; 5 Laboratoire d’ImmunoRhumatologie Moléculaire, Centre de Recherche d’Immunologie et d’Hématologie and Centre de Recherche en Biomédecine de Strasbourg (CRBS), Faculté de Médecine, Fédération de Médecine Translationnelle de Strasbourg (FMTS), Institut national de la santé et de la recherche médicale (INSERM) UMR_S 1109, Fédération Hospitalo-Universitaire OMICARE, Université de Strasbourg, Institut Thématique Interdisciplinaire (ITI) Transplantex NG, Laboratoire d’Immunologie, Pôle de Biologie, CHU de Strasbourg, Strasbourg, France

**Keywords:** Cell death and immune response, Inflammation

## Abstract

Elimination of apoptotic neutrophils by macrophages, a process called efferocytosis, is a critical step in the resolution of inflammation. Efferocytosis induces the reprogramming of macrophages towards a pro-resolving phenotype and triggers the secretion of pro-resolving factors. While mouse efferocytic macrophages are well-described, less is known about human efferocytic macrophages. Here, using RNA sequencing analysis of three different types of in vitro-derived human efferocytic macrophages, we observed a common modulation of mitochondrial metabolism-related genes in human M0, M1, and M2a-like macrophages, thus correlating with some previous results obtained in other non-human models. These results led us to identify for the first time some particular genes regulated in humans like *PLIN5* and *MTLN*. We also shed light on a mitochondrial gene (*MT-RNR2)* coding a secreted factor called HUMANIN. Mainly known for its antioxidant and neuroprotective effects, we found that HUMANIN was also associated with pro-resolving properties in human and mouse models. Indeed, HUMANIN was produced early during the resolution of inflammation in an acute peritonitis mouse model. Preventive HUMANIN administration in this model reduced leukocyte infiltration and pro-inflammatory cytokine secretion. These anti-inflammatory properties were accompanied by the early acquisition of a CD11b^low^ non-efferocytic phenotype by mouse macrophages and by an enhanced expression of pro-resolving genes including *Alox15* and *Retnla*. The ability of HUMANIN to dampen pro-inflammatory cytokine secretion was also confirmed in primary human neutrophils. Finally, HUMANIN was also detected in gingival crevicular fluids of patients suffering from periodontitis after the onset of inflammation, suggesting a role of HUMANIN in the control of inflammation. Overall, our data shed light on new aspects of efferocytosis in humans and identify the pro-resolving potential of HUMANIN. This illustrates its prospective therapeutic interest in inflammatory disorders.

## Introduction

Clearance of apoptotic neutrophils by phagocytes, a process called efferocytosis, is a critical tipping point for promoting inflammation resolution [1, 2]. Efferocytosis triggers a pro-resolving program in macrophages inducing a phenotype switch. Indeed, macrophages exhibit a tremendous capacity to reversibly switch their phenotype according to the micro-environment. Pro-resolving macrophages, generated through efferocytosis, shift their activities and acquire the ability to secrete specialized pro-resolving mediators to curb inflammation and promote tissue repair [3, 4]. Efferocytosis impairment was observed in many chronic inflammatory diseases like atherosclerosis, Alzheimer’s disease, systemic lupus erythematosus, rheumatoid arthritis or Crohn’s disease, shedding light on its crucial role in inflammation regulation [5, 6]. Therefore, modulating efferocytosis has become an innovative strategy for treating inflammatory disorders over the past years [7]. However, to achieve this goal, we need a better understanding of this process.

Transcriptomic analyses of efferocytic macrophages have highlighted the critical role of metabolic reprogramming through the remodeling of mitochondrial functions, both in human and rodent macrophages [8–12]. Mitochondria are highly versatile organelles involved in energy production, ion availability and redox balance regulation [13, 14]. These functions are crucial for the efficient execution of efferocytosis and the acquisition of a pro-resolving phenotype [8, 15–17]. To fulfill these diverse roles, mitochondria continuously sense and respond to cellular signals through the release of signaling peptides, collectively referred as mitochondria-derived peptides (MDP) or mitokines [18–20].

A prototypical example of an MDP is HUMANIN (HN), a 34-amino acid peptide, encoded by an open reading frame located within the mitochondrial 16S ribosomal RNA gene [21]. This peptide exhibits potent cytoprotective and anti-apoptotic properties across various cell types through its ability to bind membrane receptors, such as formyl peptide receptor 2 (FPR2) and the trimeric ciliary neurotrophic factor receptor alpha (CNTFR)/gp130/WSX-1 complex [22–25]. Thus, mitokine secretion is an example of how mitochondria can regulate cellular functions. But so far, no involvement of HN in efferocytosis has been shown.

While transcriptomic analyses in rodent phagocytes have provided valuable insights into the cellular reprogramming that occurs during efferocytosis [8, 15, 17], fewer studies have focused on human systems [10, 11, 26]. Indeed, interspecies differences introduce a layer of complexity, as demonstrated by disparities in arginine metabolism [26–28] or in the anti-inflammatory activity of liver X receptors [11, 26, 29, 30]. These findings underscore the critical need for studies conducted in human models to better understand the mechanisms underlying efferocytosis.

By using an unusual model of efferocytosis, based on human primary monocyte-derived macrophages (MDM) and human primary apoptotic neutrophils (PMN), which is exempt of phagocyte RNA contamination from engulfed PMN [31], we show here by bulk RNA sequencing (RNA-seq) that the upregulation of oxidative phosphorylation (OXPHOS) related genes is a hallmark of efferocytosis shared across M0, M1 and M2a efferocytic human macrophages. We shed light on the modulation of OXPHOS-related genes by efferocytosis, including those encoding the mitokine HN. We showed that HN production and liberation were stimulated by efferocytosis and we pointed out its pro-resolving abilities in in vitro human models and in in vivo and in vitro mouse models. We also detected HN in gingival crevicular fluid (GCF) from patients suffering from periodontitis after the onset of inflammation, highlighting its potential role in controlling inflammation. These results give a better overview of metabolic changes induced by efferocytosis in humans and emphasize the therapeutic potential of HN for developing innovative pro-resolving strategies.

## Results

### Transcriptomic analysis of different human efferocytic macrophage subsets highlights enhanced mitochondrial metabolism

**Fig. 1:**
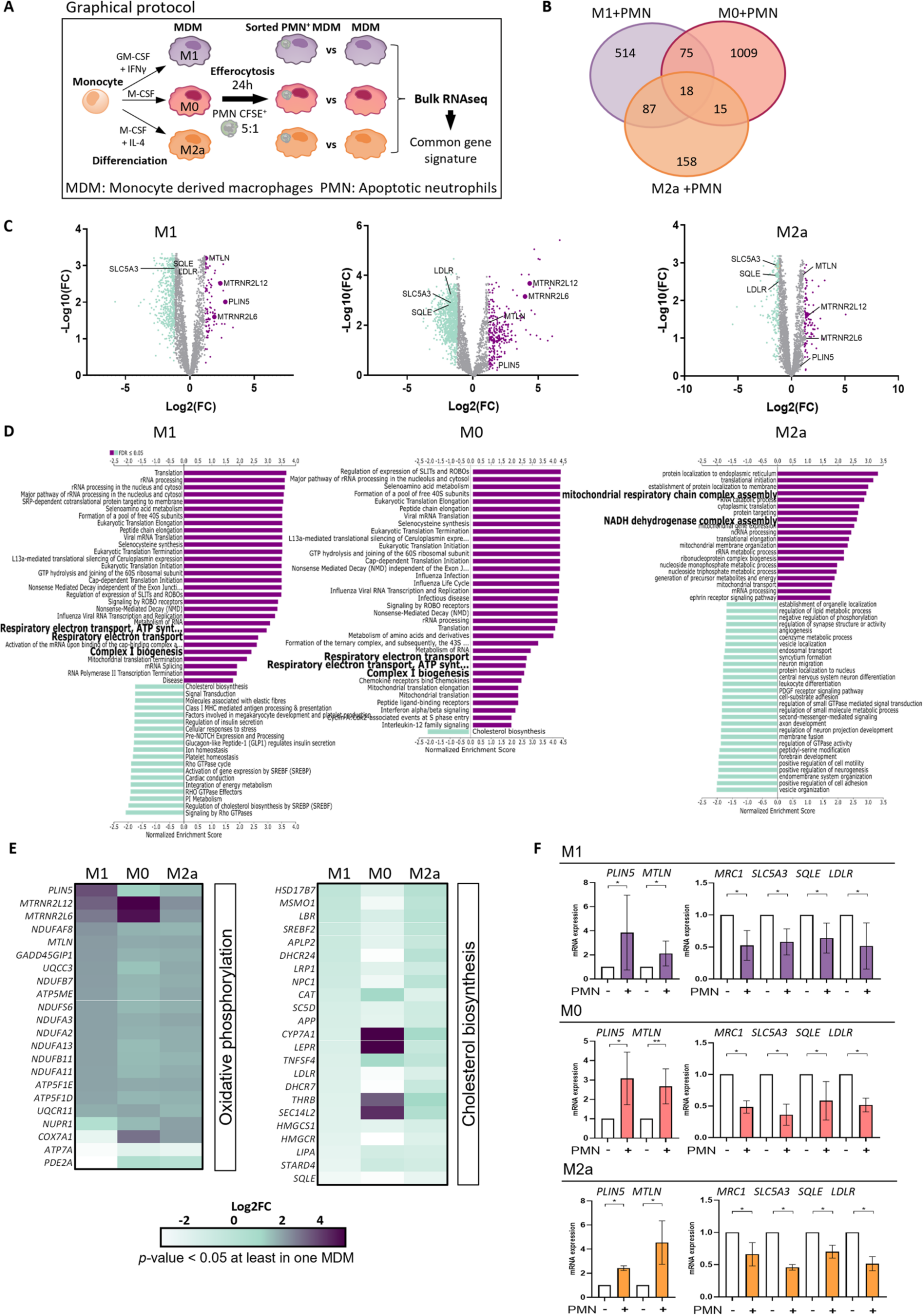
Efferocytosis induces a transcriptomic program related to mitochondrial metabolism in distinct human macrophage subsets. **A** Schematic view of a full human in vitro efferocytosis model. Human M1-, M0- and M2a-like monocyte-derived macrophages (MDM) were co-cultured with CFSE-stained human apoptotic neutrophils (PMN) for 24 h. After the removal of non-engulfed neutrophils, CFSE^+^ efferocytic macrophages were sorted by flow cytometry and used to perform bulk-RNA-seq. **B** Venn diagram representing shared and cell type-specific differentially regulated genes (DEG) in M1-, M0- and M2a-like MDM after efferocytosis. Eighteen highly modulated genes (log Fold change ± 1.2 *p*-value ≤ 0.05) were found commonly regulated in all three types of macrophages. **C** Volcano plots of DEGs in M1-, M0- and M2a-like efferocytic macrophages with a cut-off value of log2(FC = 1.2). Up- and down-regulated genes are colored in purple and green, respectively. **D** GSEA analysis of DEG after efferocytosis in M1-, M0- and M2a-like efferocytic macrophages using Reactome database. Pathways of interest were highlighted in bold. Up- and down-regulated genes are colored in purple and green, respectively. **E** Heatmaps of DEG focusing on pathways related to oxidative phosphorylation (OXPHOS) and cholesterol biosynthesis according to their Log2(FC). Compared gene lists of Reactome pathways were adapted according to information acquired by manual data mining. **F** RNA-seq validation of key genes by RT-qPCR analysis. Examples of up- and down-regulated genes in M1-, M0- and M2a-like macrophages belonging to OXPHOS, cholesterol biosynthesis or common gene signature. mRNA expression was normalized using *GAPDH* gene and relative expression was based on non-efferocytic cells for each type of MDM. Statistics: Two-tailed Student’s t-test was performed, *P  < 0.05, **P  < 0.01.

During inflammation, efferocytosis is known to induce cellular reprogramming in macrophages leading to the gain of pro-resolving functions [2, 3]. To study the changes induced by efferocytosis in human macrophages, we used an in vitro efferocytosis model consisting in the co-culture of human M0-, M1- and M2a-like MDM and PMN during 24 h that we previously described (Fig. 1A) [31]. This model was proven suitable for RNA-seq investigation, since PMN RNA is too degraded to contaminate macrophage RNA. Moreover, within this timing, spontaneous neutrophil apoptosis reached 85% after 13 h of incubation and remains around 80% at 24 h, thus avoiding any potential artifacts arising from massive necrosis [31]. Apoptotic PMN were stained with CFSE to allow us to sort and analyze only CFSE^+^ efferocytic macrophages by RNA-seq analysis. Transcriptomic analysis revealed thousands of differentially expressed genes in the three types of MDM. M0-like efferocytic macrophages exhibited the largest number of differentially regulated genes (DEG) after 24 h of efferocytosis (1117 genes, fold change ± 1.2, *p*-value ≤ 0.05) (Fig. 1B, C). Interestingly, a comparison between the three efferocytic macrophage subsets revealed 18 significant commonly regulated genes that reached a fold change threshold of ± 1.2.

To decipher the main functions regulated by efferocytosis, we then performed gene set enrichment analysis (GSEA) on each MDM subset. A summary of the up- and down-regulated pathways is shown in Fig. 1D. Several regulated pathways were observed in M1-, M0- and M2a-like efferocytic macrophages. M0-like efferocytic macrophages demonstrated only one down-regulated pathway (cholesterol biosynthesis), which was suppressed in the other two subsets (Fig. 1D, E). Inhibition of cholesterol synthesis has been previously reported [10, 11, 32]. Moreover, efferocytic macrophages shared up-regulation of genes related to OXPHOS (Fig. 1D, E). These findings are in line with some previous studies, obtained in humans and mice [8, 10]. A comparative analysis of publicly available RNA-seq datasets from human (including our dataset and reference [10] and from murine efferocytic macrophages [33–35], showed a similar pattern of OXPHOS-related genes regulation (Suppl data 1). In our study, most of these up-regulated genes encoded for respiratory chain complex proteins (Fig. 1E), except four of them, *MTRNR2L12*, *MTRNR2L6*, *PLIN5* and *MTLN* which are associated with OXPHOS and fatty acid oxidation (FAO) regulatory function [22, 36–39]. Interestingly, efferocytic M1 macrophages demonstrated the most pronounced up-regulation of those four genes compared to M0-like or M2a-like MDM (Fig. 1D, E). We validated these RNA-seq data by RT-qPCR analysis of some up-regulated (*PLIN5* and *MTLN*) and down-regulated genes (*MRC1*, *SLC5A3*, *SQLE* and *LDLR*) (Fig. 1F). Increased protein expression of PLIN5 and MTLN was also confirmed by Western Blot analysis (Suppl data 2). Altogether, these findings confirm some previous observations, related to metabolic regulation, and bring light to new efferocytosis-associated factors in human macrophages that may participate in their reprogramming. It emphasizes the impact of efferocytosis on the regulation of mitochondrial metabolism genes whatever the initial macrophage phenotype before apoptotic corpse uptake.

**Fig. 2:**
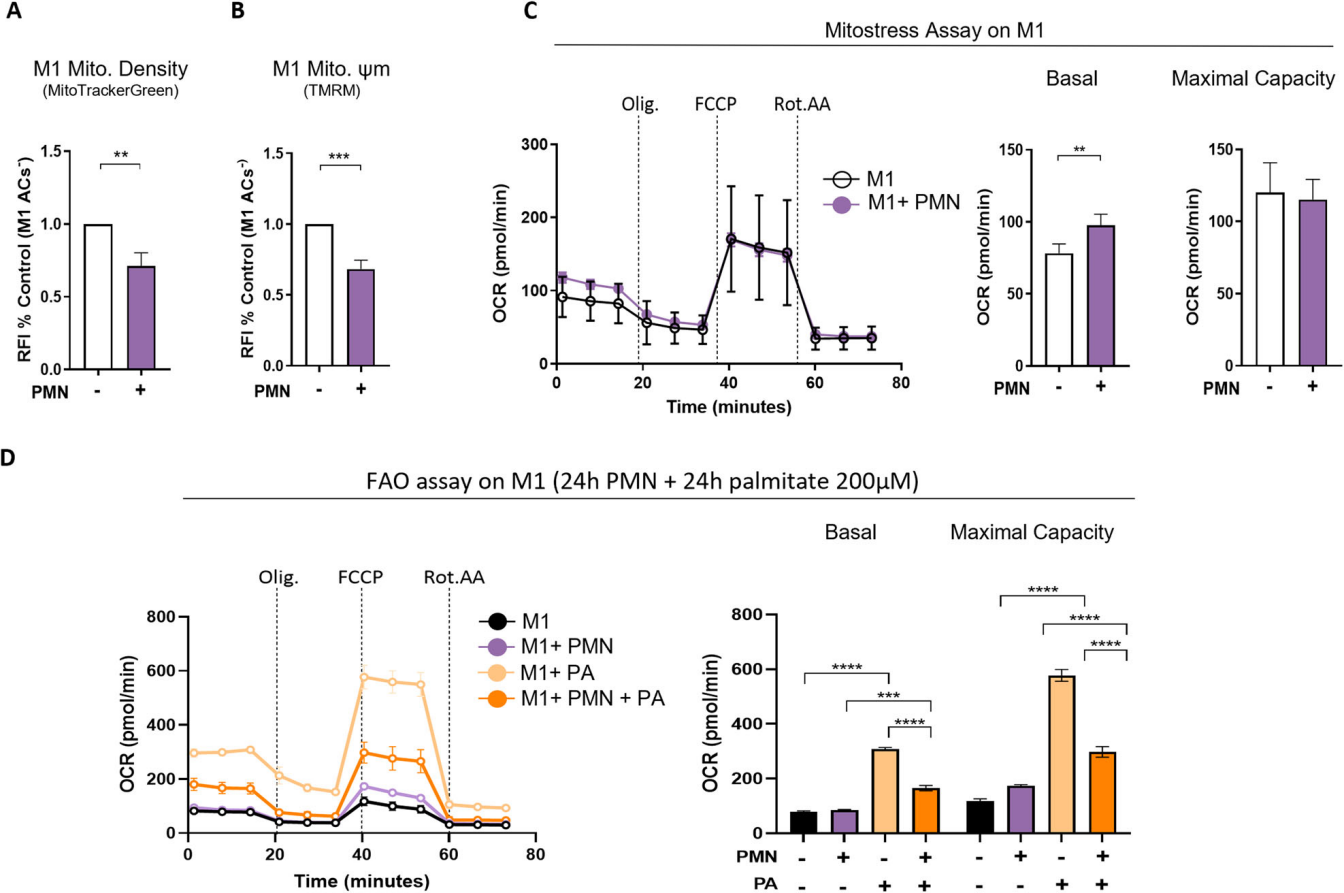
Efferocytosis reduces mitochondrial content and membrane potential, while altering respiratory activity in human pro-inflammatory M1-like macrophages. Quantification of mitochondrial density (Mito. Density) (**A**) and membrane potential (Mito. Ψm) (**B**) were assessed by flow-cytometry using MitoTracker Green FM^TM^ and TMRM, respectively. M1-like macrophages were co-incubated with CFSE-labeled apoptotic neutrophils (PMN) for 24 h, and then stained with mitochondrial dyes before analyzing by flow cytometry. Efferocytic M1-like macrophages were gated as CFSE^+^ cells. Results are expressed as Relative Fluorescent Intensity (RFI) to each non-phagocytic macrophage (M1 PMN^–^, at least *n* = 7). **C** Measurement of oxygen consumption rate (OCR) of M1-like macrophages 24 h after apoptotic neutrophil efferocytosis using the Seahorse analyzer. After efferocytosis, macrophages were submitted to sequential inhibitors treatment: oligomycin (Olig., ATP synthase inhibitor), FCCP (decoupling agent) and rotenone/antimycin A (Rot.AA, complex I and complex III inhibitors, respectively) to measure the basal and maximal respiratory capacity of the cells (at least *n* = 3). Non-efferocytic M1-like macrophages (M1) were compared to efferocytic M1-like macrophages (M1 + PMN). **D** To promote fatty acid oxidation, M1-like macrophages were incubated (or not) for 24 h with apoptotic neutrophils (PMN) and were then stimulated with palmitate (PA, 200 µM). The Seahorse assay was performed using the same inhibitors as described previously in (**C**). OCR was measured in the different groups using the Seahorse analyzer. *Statistics:* Two-tailed paired t-test and two-way ANOVA were performed according to test requirements, **P < 0.01, ***P < 0.001, ****P < 0.0001.

To further validate this mitochondrial metabolic rewiring identified by the transcriptomic analysis, changes in the dynamics and the respiration of mitochondria were investigated in efferocytic human macrophages. As efferocytic M1-like macrophages exhibited the more pronounced OXPHOS transcriptomic signature, we chose this cell type to assess functionally their metabolic changes. Macrophages were labeled with MitoTracker green FM™ and TMRM dyes to assess by flow cytometry mitochondrial density and membrane potential, respectively (Fig. 2A, B). Efferocytic M1-like macrophages exhibited a reduction in mitochondrial mass and membrane potential (Fig. 2A, B). Using the Seahorse technology, we detected an increase in basal oxygen consumption rate (OCR) in efferocytic M1-like macrophages (Fig. 2C). A previous study has reported enhanced mitochondrial respiration in human macrophages following efferocytosis [40]. Moreover, as previously demonstrated in mouse macrophages, this increase in mitochondrial respiratory capacity requires the involvement of fatty acid oxidation (FAO) in the processing of apoptotic cell-derived lipids [8]. Interestingly, we also detected in our RNA-seq data set genes involved in lipolysis, including the down-regulation of the *LIPA* gene (Suppl data 3), a gene previously characterized in murine efferocytic/pro-resolving macrophages [32] and the up-regulation of FAO-related genes *PLIN5* and *MTLN*. In light of this transcriptional signature, we assessed FAO in human efferocytic M1-like macrophages by measuring their OCR following stimulation with palmitate (200 µM) for 24 h (Fig. 2D). We showed an increase in both basal and maximal respiratory capacity in M1-like macrophages, while efferocytic M1-like macrophages exhibited a less pronounced response (Fig. 2D). To assess the content in lipid droplets, macrophages were stained with LipidTOX Red dye. This staining revealed that efferocytic M1-like macrophages exhibited more significant lipid content than M1-like macrophages non-exposed to apoptotic cells following palmitate exposure (Suppl data 4). Overall, these data functionally confirmed the establishment of mitochondrial metabolic reprogramming in human macrophages following efferocytosis, particularly affecting lipid metabolism in these phagocytes.

### In response to efferocytosis, M1-like macrophages produce HUMANIN, a mitokine that modulates inflammation

**Fig. 3:**
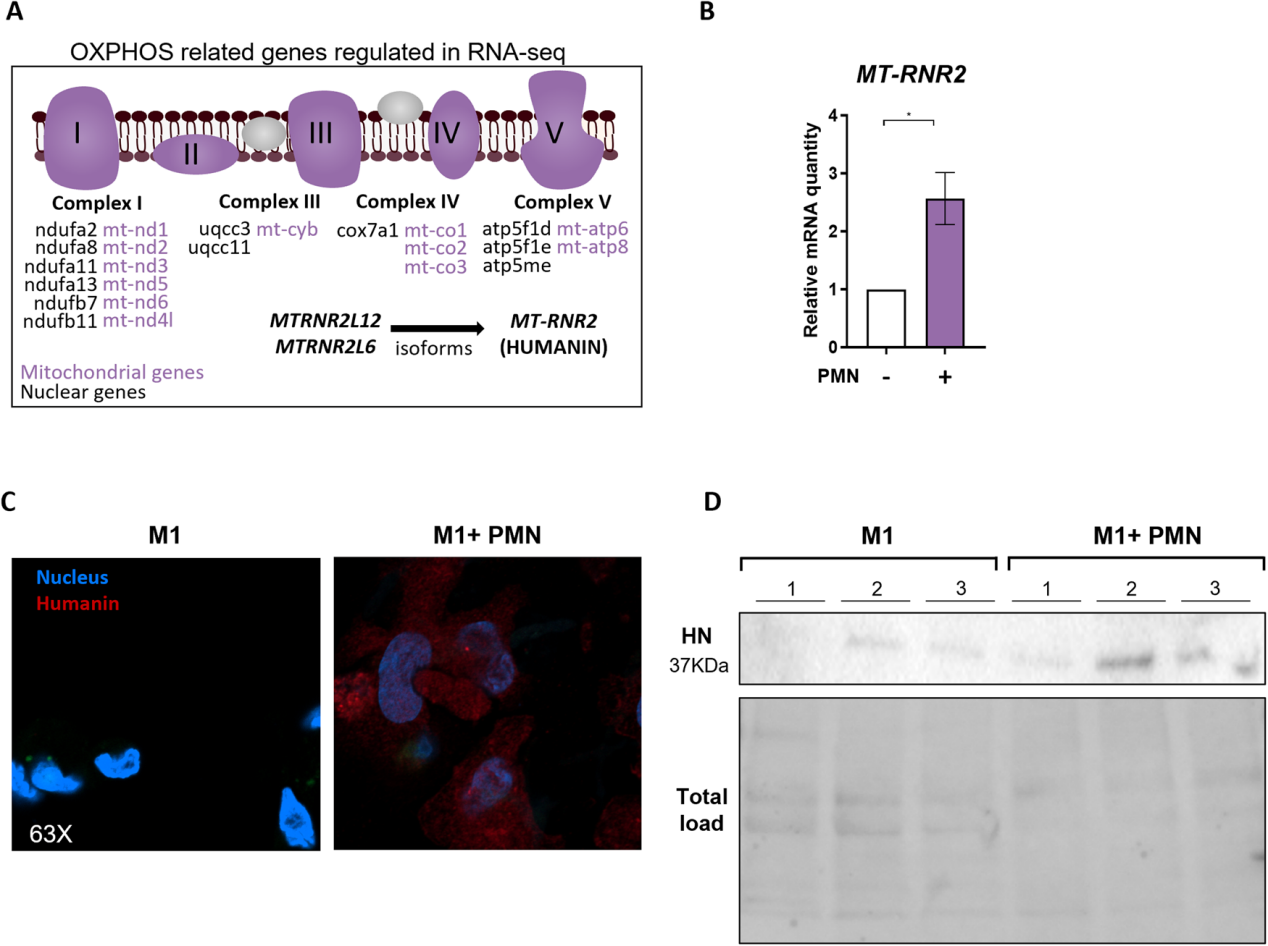
HUMANIN production is regulated by efferocytosis in humans. **A** OXPHOS-related genes found up-regulated in efferocytic M1-like macrophages in our RNA-seq analysis were mapped on the graphic according to their electron chain transport complex origin. Mitochondrial genes are written in red whereas nuclear genes are written in black. *MTRNR2L12 MTRNR2L6* genes are encoding HUMANIN isoforms. **B** Quantification by RT-qPCR of *MT-RNR2* mRNA in efferocytic (PMN^+^) and non-efferocytic (PMN^–^) M1-like human macrophages (*n* = 4). **C** Detection of HUMANIN protein in efferocytic and non-efferocytic M1-like macrophages (M1 + PMN *versus* M1, respectively) by confocal microscopy. HUMANIN is represented in red and the nucleus in blue (*n* = 3). **D** HUMANIN protein expression was assessed in efferocytic and non-efferocytic M1-like macrophages (M1 + PMN *versus* M1, respectively) by Western blotting (*n* = 3). Total protein obtained with stain-free imaging was used as a control. T-tests were used according to test requirements. *P < 0.05.

By analyzing nuclear and mitochondrial gene regulation in M1-like efferocytes, we identified a prominent upregulation of genes associated with mitochondrial complex I (NADH dehydrogenase) (Fig. 3A). Mitochondrial complex I is a primary site of reactive oxygen species (ROS) production in macrophages under inflammatory conditions [14]. In response to elevated ROS levels, mitochondria can initiate a protective metabolic adaptation known as mitohormesis [41]. This process includes the production of mitokines which promote antioxidant defense and improve mitochondrial metabolism [20]. Our attention was caught by the up-regulation of *MTRNR2L12* and *MTRNR2L6* genes in human efferocytic macrophages (Fig. 1C), which correspond to two nuclear isoforms of the mitochondrial gene *MT-RNR2* encoding for the MDP called HUMANIN (HN). Our interest focused on this mitochondrial peptide due to its ability to enhance OXPHOS activity, to regulate ROS production through mitochondrial complex I, and to exert potent cytoprotective and anti-inflammatory effects across various cell types [42–44]. Moreover, HN could interact with FPR2, a receptor also used by other pro-resolving molecules such as Lipoxins or Annexin-A1 [45, 46]. These observations led us to hypothesize that HN may derive from efferocytic macrophages to participate in the resolution of inflammation. We quantified *MT-RNR2* expression in efferocytic M1-like macrophages by RT-PCR. *MT-RNR2* mRNA expression in M1-like macrophages was enhanced after efferocytosis (Fig. 3B). This increase of genes coding HN after efferocytosis was confirmed at the protein level by confocal microscopy and Western blotting (Fig. 3C, D).

**Fig. 4:**
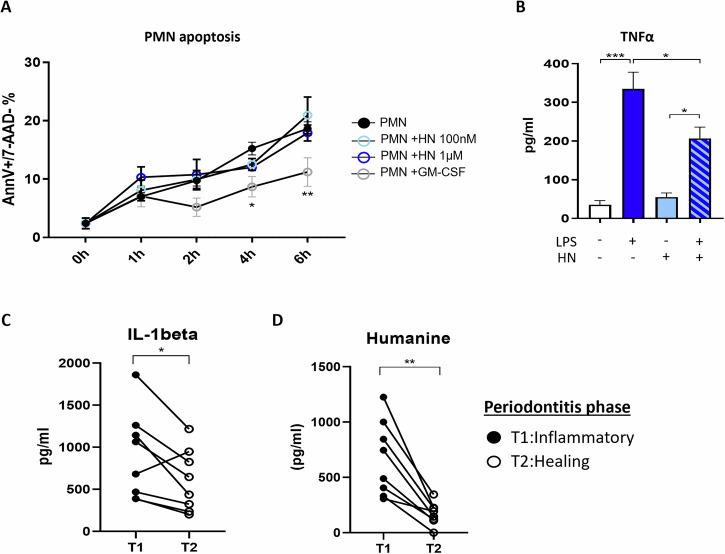
HUMANIN regulates inflammatory activities but not the viability of human neutrophils, and its level correlates with the inflammatory status of patients suffering from periodontal disease. **A** Assessment of apoptosis of PMN cultured with HUMANIN (HN, 100 nM or 1 µM) or GM-CSF (50 ng/ml). Apoptosis at the indicated time points was assessed by flow cytometry using Annexin-V (AnnV) and 7-AAD staining. Apoptotic cells were defined as Annexin-V^+^ and 7AAD^–^ cells (*n* = 4). **B** Effect of HUMANIN on TNFα secretion by human fresh PMN stimulated for 4 h by LPS (100 ng/ml). TNFα level in the supernatants was quantified by ELISA (*n* = 3). **C**, **D** Gingival crevicular fluid (GCF) was collected using strip papers on 1 or 2 sites from 8 patients suffering from periodontitis, during inflammatory (T1) and healing phase induced by mechanical treatments (T2). Proteins contained on the strip were eluted in 0.1 ml of protective buffer. IL-1β (**C**) and HUMANIN levels (**D**) (expressed as pg/ml) were measured, in the eluted GCF at T1 and T2, by microfluidic ELISA (ELLA technology) and ELISA respectively. Statistics: Two-tailed paired t-tests or two-way ANOVA were used according to test requirements. *P < 0.05, **P < 0.01, ***P < 0.001.

We next investigated the impact of HN on resolving immune processes. Induction of neutrophil apoptosis is promoted by numerous pro-resolving mediators [4, 47–49]. Given the anti-inflammatory and anti-apoptotic abilities of HN, we checked the ability of this protein to affect neutrophil activity and viability. During inflammation, various factors in the inflamed tissues ‒such as GM-CSF‒ delay neutrophil apoptosis [50]. As a control, addition of GM-CSF to human neutrophils increased their survival by 20% (Fig. 4A). However, addition of HN to neutrophils did not modulate neutrophil viability (Fig. 4A). We then assessed the ability of HN to counteract a pro-inflammatory signal in neutrophils. We showed that neutrophils treated with HN (50 µM) secreted almost 50% less TNFα after lipopolysaccharide (LPS) stimulation (Fig. 4B).

### HUMANIN correlates with the inflammatory stage of patients diagnosed with periodontal disease

Humanin has been previously used in other pathological rodent models and has shown beneficial effects [22, 51]. However, the temporal pattern of secretion during inflammation has not been explored yet. We studied HN production in human periodontitis, an inflammatory disease, associated with high oxidative stress, that can be treated only with mechanical interventions [52]. Gingival Crevicular Fluid (GCF) from affected teeth of patients was collected during the onset of inflammation (T1) and the resolution of inflammation (T2) after several non-surgical mechanical treatments. Treatment effectiveness was monitored by clinical observations and assessment of IL-1β decrease in GCF as previously described [47, 53, 54]. All patients complied with this observation except one who showed an increase in IL-1β from T1 to T2 despite clinical improvement (Fig. 4C). HN was detected in the GCF from these patients at the inflammatory stage and its level significantly decreased after mechanical treatments (Fig. 4D). This suggests that its production mainly occurs after the onset of inflammation and oxidative damage, when excessive inflammation needs to be controlled, and it then decreases during the gingival healing phase, when inflammation is already shut down.

### HUMANIN decreases leukocyte recruitment and activation during inflammation in a model of peritonitis

**Fig. 5:**
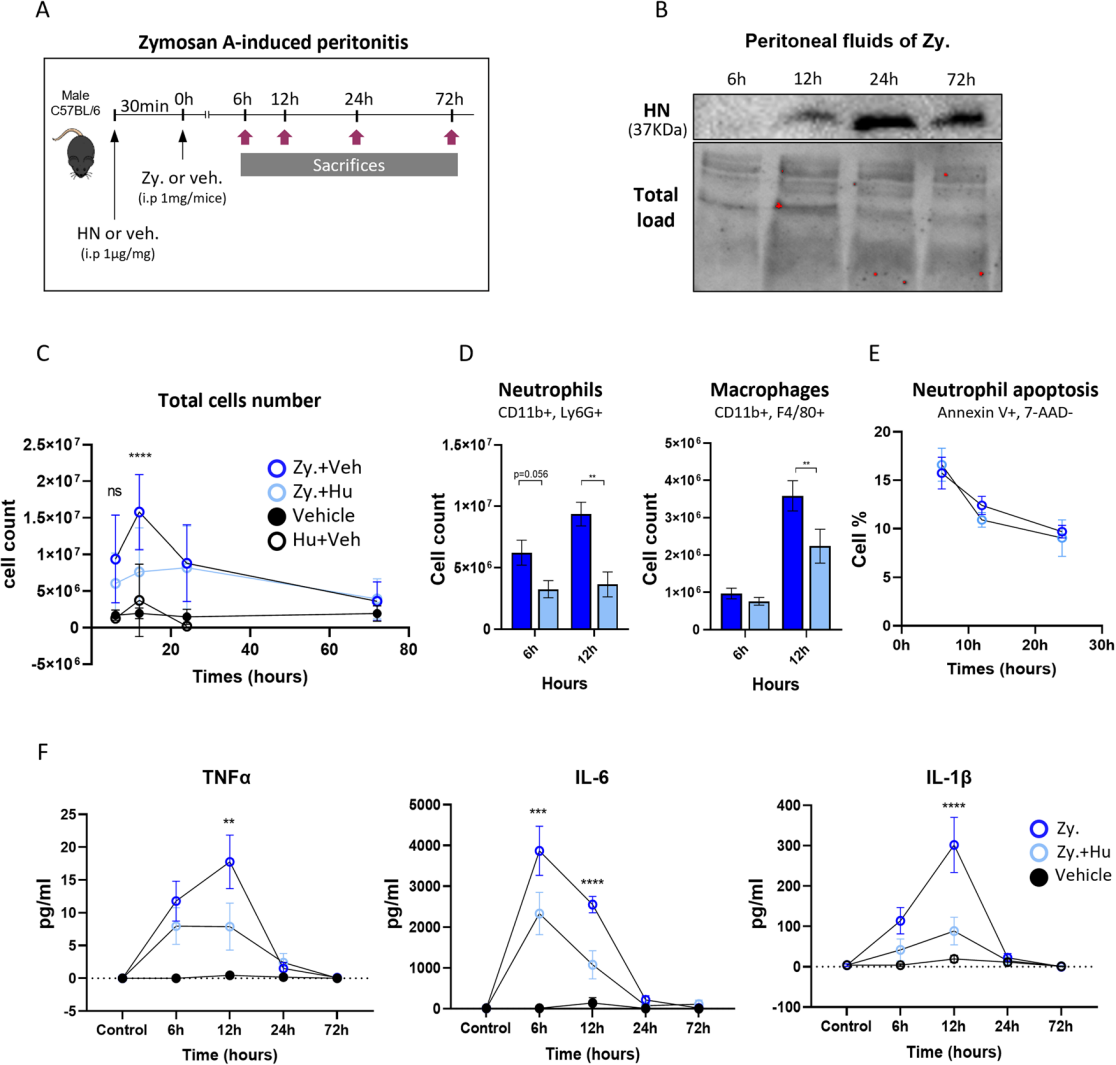
Humanin decreases leukocyte recruitment and activation in a murine peritonitis model. **A** Peritonitis was induced in C57/Bl6 mice by intraperitoneal (i.p.) injection of 1 mg zymosan-A (Zy.) resuspended in PBS (open dark blue circles) or vehicle PBS (Veh., black circles). HUMANIN (Hu, 20 µg in PBS) or vehicle (Veh) was injected i.p. 30 min before zymosan-A (Zy.+Hu., open light blue circles *vs*. Hu.+Veh., open circles, respectively). Peritoneal washes were performed at different time points (6 h,12 h 24 and 72 h) and cells were used for different analyses. **B** Western blot analysis of mouse Humanin (HN) in cell-free peritoneal fluid at 6 h, 12 h, 24 h and 72 h after zymosan-A (Zy.) injection. Total protein obtained with stain-free imaging was used as a control. (*n* = 3). **C** Total cell count in peritoneal fluid was performed manually using Trypan blue staining. **D** Flow cytometry was used to assess number of neutrophils Ly6G^+^, F4/80^−^) and myeloid cells (Ly6G^−^, F4/80^low/High^) contained in the peritoneal fluid at the different indicated time points. **E** Neutrophil viability in the peritoneal fluid at the indicated time points was assessed by flow cytometry using Annexin V and 7-AAD. **F** After zymosan-A injection, the peritoneal cavity of the mice, treated with HUMANIN or not, was flushed at the indicated time points with PBS and TNFα, IL-6 and IL-1β levels in the recovered fluids were measured by ELISA. Statistics: Two-way ANOVA and two-tailed paired t-test were used according to test requirements. Results are displayed as mean ± SEM (*n* = 10–14 mice), *P < 0.05, **P < 0.01, ***P < 0.001, ****P < 0.0001.

To study more precisely the kinetics of HN secretion and its effect during the inflammation process, we moved to an in vivo inflammatory mouse model of zymosan-A-induced peritonitis (Fig. 5A). This model corresponds to a well-established experimental model of acute self-resolving inflammation. The time course of the major cellular and molecular events of the acute and resolution phases was set up in previous works [33, 55–58]. Neutrophil infiltration and inflammatory cytokine secretion peak at around 12 h after intraperitoneal zymosan-A injection (1 mg) while they undergo apoptosis, during the onset of the resolution phase, at around 12-24 h. To monitor mouse HN secretion, Western blot analysis was performed on the cell-free peritoneal exudates obtained from 6 to 72 h after peritonitis induction. HN was not detected at the onset of inflammation at 6 h but it then progressively accumulates in the peritoneal fluid with a peak at 24 h. Thereafter, the amount of HN slightly decreased at 72 h (Fig. 5B). These results suggest that HN secretion occurs at the end of the acute inflammatory phase. Given these results, we then wanted to determine the potential regulatory role of HN in the inflammatory process. We monitored the magnitude and duration of inflammation in the context of preventive recombinant HN administration (Fig. 5A). Intraperitoneal injection of HN, 30 min before zymosan-A, reduced by about two-fold the total number of leucocytes in the peritoneal cavity at 12 h (Fig. 5C). Then, leucocyte number declined 24 h after inflammation induction as the peritonitis resolved (Fig. 5C). As a control, HN was also injected alone and did not trigger any leucocyte recruitment. We then characterized the nature of these recruited cells by flow cytometry and showed that this decrease concerned neutrophils and monocytes/macrophages at 12 h (Fig. 5D). Annexin V and 7-AAD staining of peritoneal cells showed a similar percentage of detectable apoptotic neutrophils from 6 to 24 h (Fig. 5E). We further investigated whether the levels of pro-inflammatory cytokines were modulated by HN. Peritoneal exudates of mice receiving HN showed reduced TNFα, IL-6 and IL-1β levels at 6 h with the largest decrease being observed at 12 h (Fig. 5F). Overall, these results indicate that HN can reduce directly or indirectly the recruitment of immune cells and dampen the secretion of pro-inflammatory mediators in a model of mouse peritonitis.

### HUMANIN administration increases pro-resolving CD11b^low^ macrophages

**Fig. 6:**
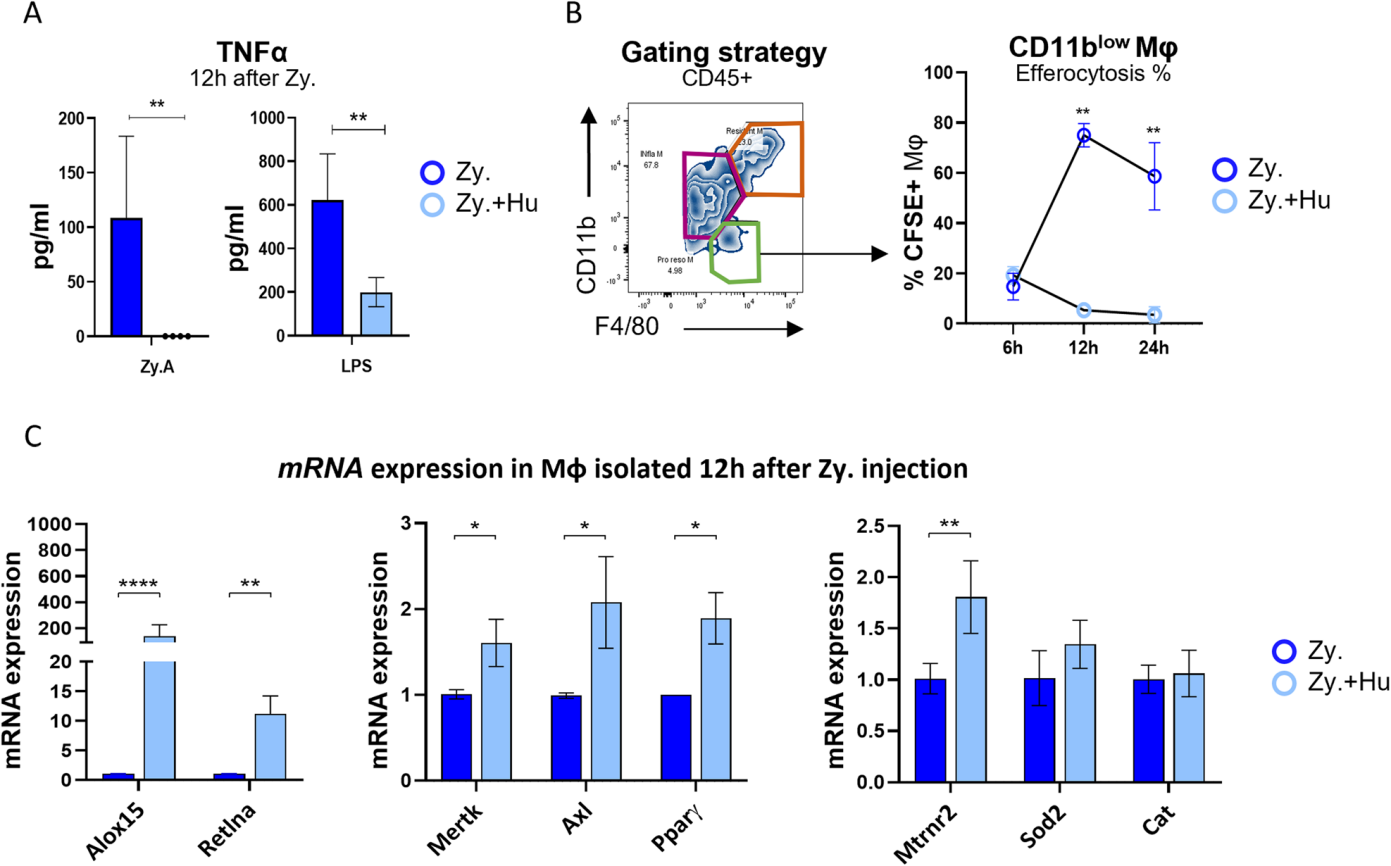
HUMANIN administration increases pro-resolving CD11b^low^ macrophages. **A** Peritoneal macrophages from mice that received zymosan-A alone (dark blue bars) or with HUMANIN (light blue bars) were collected from mice and isolated by adherence 12 h after zymosan-A injection. For the macrophage restimulation assay, zymosan-A (100 µg/ml) or LPS (100 ng/ml) were added during 16 h. TNFα protein level in cell supernatant was measured by ELISA (*n* = 4). **B** Efferocytosis of CD11b^low^ pro-resolving macrophages at different time points were assessed ex-vivo. Macrophages were collected at 6 h, 12 h and 24 h from mice with peritonitis and mixed with CFSE^+^ apoptotic thymocytes for 45 min. Non-engulfed thymocytes were removed before immuno-staining and flow cytometry analysis. Efferocytosis rates of F4/80^+^, CD11b^low^ macrophages (green square) from mice that received zymosan-A alone (open dark blue circles) or with HUMANIN (open light blue circles), were determined according to the percentage of CFSE^+^ macrophages. **C** mRNA expression of genes considered as pro-resolving genes (*Alox15, Retlna*), or involved in efferocytosis (*Mertk, Axl* and *Pparγ*) as well as antioxidant genes (*Mt-rnr2*, *Sod2* and *Cat*) in phagocytes from peritoneal cavity 12 h after injection of zymosan-A ± Humanin (Zy., open dark blue vs. Zy.+Hu, light blue). Of note, the *Mt-rnr2* gene encodes Humanin. Statistics: Two-way ANOVA and two-tailed paired t-test were used according to test requirements. Results are displayed as mean ± SEM (*n* = 10–14 mice), *P < 0.05, **P < 0.01, ***P < 0.001, ****P < 0.0001.

To decipher if HN could directly impact macrophage pro-inflammatory cytokine secretion, we isolated macrophages from the peritoneal exudate at 12 h (*i.e*., the peak of inflammation) by adherence and stimulated them with zymosan-A or LPS. Macrophages exposed in vivo to HN showed an impaired secretion of TNFα after in vitro restimulation by both pro-inflammatory stimuli (Fig. 6A). As HN seems to dampen macrophage inflammatory responses, we checked if the protein could promote the acquisition of a pro-resolving phenotype profile in these phagocytes. Mouse peritoneal pro-resolving macrophages could be identified as F4/80^+^/CD11b^low^ cells with low efferocytic capacity, a phenotype called “satiated” [55, 59]. CD11b^low^ macrophages were detected 12 h after zymosan-A administration and then progressively increased over time (Suppl data 5). Administration of HN before zymosan-A did not significantly modify the percentage of this subset at the time of resolution (mean: 32.2 ± 4.3% of F4/80^+^ CD11b^low^ macrophages in mice treated with HN *vs*. 39% ± 4.6% at 24 h). We then measured ex vivo the efferocytosis rate of F4/80^+^/CD11b^low^ macrophages. Peritoneal macrophages were isolated at 6, 12 and 24 h from mice receiving zymosan-A, treated or not with HN, and then fed with apoptotic thymocytes labeled with CFSE. F4/80^+^/CD11b^low^ macrophage from mice treated with HN showed a strikingly lower efferocytosis rate from 12 to 24 h compared to those that only receive zymosan-A (Fig. 6B), suggesting that HN promotes a faster acquisition of the satiated state. This suggests that HN promotes the resolving phenotype. To further characterize the phenotype of these macrophages, we performed RT-qPCR using macrophages extracted 12 h after zymosan-A injection. We studied HN (*Mt-rnr2)* and other genes associated with mouse pro-resolving macrophages *(i.e*., *Alox15, Retlna, Mertk, Axl, Pparγ*) or with antioxidant properties (*i.e., Sod2, Cat*) [59–62]. Peritoneal macrophages from mice injected with HN showed an increased expression of all these genes in macrophages, except those coding for antioxidant proteins (Fig. 6C). These results suggest that HN administration accelerates the acquisition of a pro-resolving profile in macrophages. Overall, these results showed that HN modulates the inflammatory reaction by promoting inflammation resolution through anti-inflammatory effects and pro-resolving phenotype promotion.

## Discussion

In this work, we performed a global transcriptomic analysis of efferocytic macrophages to unravel new factors involved in the resolution of inflammation in humans. Whereas most studies on efferocytosis rely on non-human models and focus on the early events occurring during the first hours post-efferocytosis [8, 9, 12], we adopted a different approach by performing RNA-seq analysis at a late stage (24 h) in a full human in vitro efferocytosis model using three different types of efferocytic MDM. This model, which is the closest in vitro model to human physiology currently available, was already shown to be a suitable model for RNA-seq studies, due to the absence of phagocyte RNA contamination by engulfed apoptotic neutrophils [31]. Within this timing, we aimed to capture more stable transcriptional changes associated with the acquisition of a pro-resolving phenotype by efferocytic macrophages. We also postulated that key changes may be shared across different types of efferocytic macrophages to reprogram them toward a converging resolving phenotype. Our results showed shared alterations of mitochondrial and cholesterol biosynthesis pathways in the three types of efferocytic macrophages (M0, M1, or M2a). These results are in line with previous works carried out at earlier timings on human and murine efferocytic macrophages [8, 10, 11, 55, 63], indicating that these changes are stable and thus essential for efferocytic macrophages. Our findings suggest that efferocytosis affects, whatever the initial type of macrophage, the mitochondria dynamics in humans to optimize their respiratory capacity. The scale of this reorganization seems related to the relative proximity of the initial macrophage phenotype to the resolving phenotype.

In our RNA-seq data, we noticed the strong up-regulation of *MTRNR2L12* and *MTRNR2L6* genes that are related to the HN gene *(MT-RNR2)*, which encodes for a mitohormesis-associated protein. Previous reports showed that HN can modulate mitochondrial functions and possesses antioxidant properties, as well as anti-inflammatory potential [21, 25, 64, 65]. Several research groups have demonstrated the beneficial effects of HN and its analogs in various pathological settings, without reporting detectable adverse effects [66–69]. While these results demonstrate the therapeutic interest of this mitokine, there is limited data available on the mechanisms by which HN regulates the immune system. We showed here that HN could directly exert anti-inflammatory functions on human primary neutrophils without affecting their viability.

In a model of self-resolving peritonitis, we showed that HN production begins during the late acute phase of inflammation and peaks during resolution, a timing suggesting its involvement in pro-resolving processes. Preventive HN administration reduced leukocyte recruitment and pro-inflammatory cytokines in the peritoneal cavity. Moreover, HN-treated mice exhibited early induction of macrophages with a pro-resolving phenotype. The direct impact of this mitokine on macrophage efferocytosis or polarization was not addressed in this work and remains to be deciphered. Nonetheless, the effects of HN likely involve particular interactions with receptors (e.g., FPR2), which are critical to control oxidative stress and orchestrate inflammation resolution [22, 46, 70, 71].

Oxidative stress is a key feature of periodontal inflammation [52]. HN production correlates with the active inflammatory phase during periodontal disease suggesting that HN fulfills its main action early in the reaction. This hypothesis is strengthened by the data obtained in our mouse model, showing that HN is secreted after the onset of inflammation and during the early timings of the resolution phase. It was previously shown that the HN analog, S14G-Humanin, can reduce the inflammatory response of human dental pulp cells in response to LPS stimulation [72]. This suggests a potential protective role of HN against deleterious inflammatory bacterial reactions. Pro-inflammatory LPS activity is closely associated with dysbiosis of the oral microbiota, a hallmark of periodontal diseases [73, 74]. While no direct link between HN and microbiota regulation has been established so far, it is intriguing to note that FPR2, one of HN receptors, has been implicated in gut microbiome regulation [75]. Considering these results and the pro-resolving abilities of HN demonstrated in this study, we strongly believe that HN secretion is crucial to control periodontitis-induced inflammation.

Further studies are needed to elucidate its impact when administered during different phases of inflammation and to identify additional properties in other immune cells and other sources of HN production beyond macrophages.

This work highlights mitochondrial peptide signaling as pivotal elements in the macrophage-mediated resolution of inflammation in humans. Mitochondrial peptide regulation after efferocytosis should be better considered regarding the therapeutic potential of these molecules to treat inflammatory disorders.

## Materials and methods

### Mouse experiments

Eight- to 10-week-old C57Bl/6 male mice were purchased from Janvier-Lab (Le Genest-Saint-Isle, France) and were housed in filter-top cages with freely available food and sterile water at the Franche-Comté University Animal facility. The number of animals for each experiment was determined based on previous experience with the model system. Each mouse of different groups was randomly allocated in cages. The studies were nonblinded.

### Self-resolving zymosan-A-induced peritonitis

Humanin was purchased from Eurogentec (ref: AS-60886). Zymosan-A-induced peritonitis was induced in male C57Bl/6 mice. Mice (*n* = 4–6/per group/per experiment) received an intraperitoneal injection of either zymozan-A (38.5 μg/g), HUMANIN (0.77 µg/g in PBS), HUMANIN plus zymozan-A or PBS alone. HUMANIN were injected 30 min prior to zymosan-A. Mice were sacrificed at the indicated time points (6, 12, 24, and 72 h). Peritoneal cavities were washed with 2 ml of cold PBS. Lavage fluids were separated from cells by centrifugation and used for the analysis of both HUMANIN and cytokine levels by Western blot analysis and/or ELISA. Recovered cells were resuspended in RPMI medium and total living cell count was measured manually using Trypan blue to exclude dead cells. Cells were further analyzed using flow cytometry, ex-vivo efferocytosis assay, cytokine assays or RT-qPCR analysis. For ex-vivo efferocytosis assay, thymocytes were extracted from naïve C57Bl/6 mice. Mice exhibiting blood contamination in the peritoneum post-sacrifice were excluded from the analysis.

### Human blood samples

All primary cells were prepared from leukocyte-platelet concentrates and cytapheresis kits obtained from healthy donors. All the methods used for purification, culture and cell phenotyping of human cells were described previously [31].

### Cell purification and culture of primary human and mouse cells

For human monocyte-derived macrophage (MDM) generation, peripheral blood mononuclear cells (PBMCs) were isolated by Ficoll density gradient separation (Eurobio). Monocytes were then isolated from PBMCs by CD14^+^ cell selection (human CD14+ MicroBeads, Miltenyi Biotec) according to manufacturer’s recommendations. Purity was assessed by CD14 labeling with CD14-PE-CF594 antibody (BD Biosciences) by flow cytometry and was at least 95%. Primary CD14^+^ monocytes were differentiated into MDM in complete RPMI 1640 GlutaMAX™ (ThermoFisher) supplemented with 10% fetal bovine serum (Gibco), 1X penicillin/streptomycin (Thermo-Fisher), 1X non-essential amino acid (Sigma), 1X Sodium Pyruvate (Sigma) and various cytokines at 50 ng/ml (premium grade, Miltenyi Biotec) depending on the desired MDM subtypes. GM-CSF and IFN-γ were used to generate M1-like macrophages, M-CSF for M0-like and M-CSF and IL-4 for M2a-like macrophages. Cells were kept at 37 °C, 5% CO_2_ for 7 days to allow their differentiation into macrophages and one volume of medium was added at day 3–4. Macrophage phenotypes were routinely verified by cytometry.

Human primary neutrophils (PMN) were isolated from fresh leukocyte-platelet concentrates using Ficoll density gradient separation (Eurobio) followed by sedimentation in a 3% dextran solution (Sigma). The upper layer containing neutrophils was collected and washed in PBS. The remaining red blood cells were lysed by incubation of the pellet in a hypertonic solution (NH4Cl (0.077 M), EDTA (0.633 mM), KHCO3 (5 mM)) for 10 min. Lysis was stopped by the addition of PBS and neutrophils were centrifuged and counted. Neutrophil purity was assessed by flow cytometry using CD16-APC (Sony) and CD15-FITC (Sony) antibodies and purity was at least of 80%. Purified neutrophils were incubated at 5 million cells per ml in complete RPMI culture medium overnight at 37 °C to induce spontaneous apoptosis. Apoptotic rate was routinely confirmed using Annexin-V and 7-AAD staining (eBioscience) by flow cytometry. If living neutrophils are needed, experiments were proceeded directly after isolation.

For murine thymocyte isolation, thymus from different 8 to 10-week-old C57Bl/6 mice were extracted and dissociated. Single-cell suspension was obtained by filtration through a 40 µm strainer. Thymocytes were collected and red blood cells were lysed using a hypertonic solution (NH4Cl [0.077 M], EDTA [0.633 mM], KHCO3 [5 mM]). Lysis was stopped by PBS addition and cells were centrifuged and resuspended in complete RPMI medium (GIBCO). Apoptosis was induced by 35 X-Gray irradiation followed by 4 h at 37 °C and overnight 4 °C incubation. Apoptosis was controlled by flow cytometry.

### Human subjects and GCF sample collection

Gingival crevicular fluid (GCF) was collected from eight patients suffering from periodontitis at the inflammatory stage (T1_infla_) and after periodontitis resolution (T2_reso_; 2 months after the first mechanical treatment). Paper strips were placed between teeth in order to receive the GCF. Paper strips were placed into a sealed Eppendorf tube containing 0.1 ml PBS with proteinase inhibitors (Thermofisher). The samples were directly frozen at −20 °C until transfer for analysis.

### Flow cytometry

For in vitro experiments, macrophages were removed from the wells using a stripper after 20 min of EDTA (Invitrogen) (10 mM) in PBS incubation. Cells were washed and incubated with a Fcγ receptor blocking agent (ThermoFisher) and viability dyes. Cells were washed and stained with the appropriate fluorochrome-conjugated monoclonal antibodies diluted in PBS with 3% BSA (PAN BIOTECH) (wt/vol) at +4 °C. Cells were washed and analyzed on a Sony SP6800 Spectral Analyzer for human cells. For neutrophil apoptosis assay, cells were stained with FITC-conjugated Annexin-V and 7-AAD (BD Pharmingen) according to the manufacturer’s recommendations followed by antibody staining in PBS with 3% wt/vol BSA, before analyzing by flow cytometry.

For in vivo murine experiments, extracted cells were stained with Fcγ receptors blocking agent and viability dyes directly followed by fluorochrome-conjugated monoclonal antibody staining.

### Efferocytosis assay

For in vitro efferocytosis assay, apoptotic human neutrophils or murine thymocytes were stained with 5 µM CFSE or 2.5 µM Cell trace Far Red dye (Thermo Fisher) according to manufacturer’s recommendations. Medium from MDM or mouse peritoneal macrophage cultures was replaced by serum-free Xvivo15 medium (LONZA) and labeled apoptotic cells were added to human and murine macrophages at a 5:1 ratio (*i.e*., 5 apoptotic cells for one macrophage) for the indicated time. At the end of the co-culture, apoptotic cells were removed from adherent macrophages by two PBS washes. Flow cytometry was used to determine the efferocytic index defined as the percentage of CFSE^+^ or Far Red^+^ positive cells within the live CD206^+^ human and F4/80^+^ CD11b^low/high^ murine macrophages.

### NGS library preparation and RNA sequencing

Human monocyte-derived M1- M0- and M2a-like macrophages and CFSE^+^ apoptotic neutrophils were co-cultured for 24 h. Phagocytic macrophages (CD206^+^ and CFSE^+^) were then sorted using the SH800S Sony Cell sorter. Total mRNA of CFSE^+^ macrophages and macrophages cultured without neutrophils was isolated using a Nucleospin RNA kit (MACHEREY-NAGEL) according to the manufacturer’s protocol. RNA quality control was performed using Experion™ Automated Electrophoresis System according to the manufacturer’s instructions.

### RNA sequencing data processing, differential expression analysis and GSEA analysis

Sequenced raw reads were aligned following the STAR method using the human GRCh38 Genome reference and the sequences were annotated with BioMart. Differential gene expression analysis was performed by using RSEM/DESeq/edgeR, and Limma-Voom pipeline. Statistically differentially expressed genes were selected following log2FC + >1.2 and adjusted *p*-value (*q*-value) ≤ 0.05 and visualized with volcano plots. The main analysis was conducted without mitochondrial genes while they were included in the analysis described in supplementary data. GSEA was performed using the website tool WebGestalt. Reactome, KEGG and GO Database with manual data mining were used to analyze regulated pathways. RNA sequencing was performed on 2B2S bioinformatics platform at Rennes (France) and raw data were processed by the PEAT²t platform (Franche-Comté University, France). The RNA-seq data are available at the European Nucleotide Archive under accession number PRJEB37205.

### Seahorse assay

For assessing oxygen consumption rate (OCR) after efferocytosis, macrophages were plated on XF96 cell culture microplate coated with poly-lysine D (SIGMA) at a concentration of 60 000 cells/per well. For mitochondrial stress assay, experiments were conducted in XF assay medium containing 25 mM glucose, 2 mM L-glutamine, and 1 mM sodium pyruvate according to manufacturer instructions (Agilent Technologies). For Fatty acid oxidation assay (FAO assay) after efferocytosis, macrophages were incubated with 200 µM of palmitate (Cayman) for 24 h in X-vivo15 medium before being plated and analyzed on XF96 extracellular flux analyzer (Agilent Technologies). When indicated, chemical inhibitors were injected in the following order: oligomycin (ATP-synthesis inhibitor, 1.5 μM), carbonyl cyanide 4-(trifluoromethoxy) phenylhydrazone (FCCP; ATP synthesis uncoupler 1.5 μM), and rotenone (complex I inhibitor, 100 nM) and antimycin A (complex III inhibitor, 1 μM) (Agilent Technologies). For FAO assay, etomoxir (40 µM) was added to macrophages in a XF96 microplate 30 min before the beginning of the experiment to block fatty acid entry into the mitochondria. Basal OCR, maximal and spare capacity were calculated using Wave Desktop software (Agilent Technologies). The products used for Seahorse analysis are described in Table 1.

**Table 1:** List of products used in Seahorse experiments.

Product name	Supplier	Reference
Seahorse XFe96 Analyzer	Agilent	411009
D-Glucose solution 45% in H_2_O	Sigma	G8769
Sodium Pyruvate solution 100 mM	Sigma	S8636
L-Glutamine solution 200 mM	Sigma	G7513
XF DMEM Medium pH 7,4–500 ml	Agilent	103575-100
Cell Culture Microplate V3-PS TC-Treated	Agilent	101085-004
Seahorse XF Cell Mito Stress Test kit	Agilent	103015-100
Poly-D-Lysine 0,1 mg/mL	GIBCO	A3890401

### Mitochondrial analysis

Mitochondrial density staining and mitochondrial membrane potential were measured by incorporation of MitoTracker Green FM^TM^ (MTG) and TetraMethylRhodamine Methyl Ester Perchlorate (TMRM, a cell-permeant, cationic, red-orange fluorescent dye that is readily sequestered by active mitochondria) (Thermo Fisher Scientific) into living macrophages. After efferocytosis, PMN were washed and macrophages were incubated with pre-heated PBS with 50 nM TMRM or 50 nM MTG for 20 min. Cells were washed twice with preheated PBS and stained according to the flow cytometry protocol described above before analysis on a cytometer. Mitochondrial dye intensity was measured in live CD206^+^ efferocytic macrophage population.

### Cytokine measurement

Human IL-6 and TNFα ELISA kits were purchased from R&D Systems. HUMANIN quantification from cell cultures and in GCF samples was performed using Human HUMANIN ELISA Kit (#XPEH9261, XpressBio). Human IL-6, TNFα, IL-1β were quantified using multiplexed ELLA cartridges (R&D Systems). ELLA microfluidic analyzer (Biotechne) was used to assess cytokines in small samples arising from GCF. Mouse IL-6 and TNFα ELISA plates were purchased from Biolegend, while IL-1β and CXC10 were detected by using commercially available kits (R&D Systems). ELISA were conducted according to the manufacturer’s instructions.

### RT-qPCR analysis

At the indicated time points, macrophages were washed with PBS and then total RNA was extracted using NucleoSpin RNA kit (MACHEREY-NAGE) and quantified by spectrometry (Nanodrop) at 260 and 280 nm. Then, cDNA was synthesized using RevertAid H Minus First strand cDNA kit (Thermo Fischer) according to the manufacturer’s instructions. Quantitative RT-PCR was performed using a 7500 Real-Time PCR system (Applied Biosystems) and SYBR Green (SsoAdvanced Universal SYBR Green Supermix (Biorad). The primers used are described in Table 2.

**Table 2:** List of primers used in Q-PCR experiments.

Species	Primers	Sequence Forward	Sequence Reverse
Human	*MRC1*	GCAGTGGGATCACTTTCACA	ACGAGGAGGTAAAGGGCAAT
Human	*MTLN*	CGCTACTTGGACTGGAGGAA	AGCAACACTCCAGGAAGAGC
Human	*LDLR*	AATGCTTGGACAACAACGGC	CACTTGTAGCCACCCTCCAG
Human	*PLIN5*	TCCGGATCTCTGACCACCTT	GGCCTGAATAGGGTTCGAGG
Human	*SLC5A3*	GCTGGCTTTGTTCTTGGAGC	GTGGGAGGTGGTGTGAGAAG
Human	*SQLE*	CAGATGATTCCCTGCATCAA	ACACGGCATAGATTGCAACA
Human	*MT-RNR2*	TACCCCGCCTGTTTACCAAA	GGGCAGGTCAATTTCACTGG
Human	*GAPDH*	TTGCCATCAATGACCCCTTCA	CGCCCCACTTGATTTTGGA
Mouse	*Alox15*	CTCTCAAGGCCTGTTCAGGA	GTCCATTGTCCCCAGAACCT
Mouse	*Retnla*	CCAATCCAGCTAACTATCCCTCC	CCAGTCAACGAGTAAGCACAG
Mouse	*Mertk*	CAGGGCCTTTACCAGGGAGA	TGTGTGCTGGATGTGATCTTC
Mouse	*Axl*	ATGGCCGACATTGCCAGTG	CGGTAGTAATCCCCGTTGTAGA
Mouse	*PPARγ*	TTTTCCGAAGAACCATCCGATT	ATGGCATTGTGAGACATCCCC
Mouse	*Mt-rnr2*	AATTTCGGTTGGGGTGACCT	AGGATTGCGCTGTTATCCCT
Mouse	*Sod2*	CAGACCTGCCTTACGACTATGG	CTCGGTGGCGTTGAGATTGTT
Mouse	*Cat*	GTGGTTTTCACTGACGAGATGGCA	TCGTGGGTGACCTCAAAGTATCC
Mouse	*Gapdh*	AGGGTGGAGCCAAAAGGGT	GAGCCCTTCCACAATGCCAAA

### Antibodies

Anti-human antibodies used for flow cytometry were CD14-PE-CF594 (Biosciences, 562335), CD15-FITC (Sony, 2109520), CD16-APC (Sony, 2110060), CD80-BV421 (Sony, 2126110), CD163-AlexaFluor647 (BD Biosciences, 562669), CD206-BV421 (BD Biosciences, 564062), CD206-BB515 (BD Biosciences, 564668), hMER-PE (Sony, 2438040).

Anti-murine antibodies used were CD45-BV711 (BD Biosciences, 563709, CD11b-APC-Cy7 (BD Biosciences, 557657), F4/80-BV421 (D Bioscience, 565411) and Ly6g-BV605 (BD Biosciences, 5663005).

### Western blot analysis

Total protein was extracted from macrophages using RIPA lysis buffer (Millipore, 20-188) containing protease (Roche, 5892988001) and phosphatase inhibitors (Roche, 4906845001). Protein concentration was quantified using a BCA assay kit (Pierce,23235). Protein samples or peritoneal liquids were separated by SDS-PAGE, transferred to PVDF membranes, and blocked with 5% non-fat milk. Membranes were then incubated overnight at 4 °C with the following primary antibodies anti-HUMANIN (NovusBio, 56877SS), anti-PLIN5 (NovusBio, 60509SS), anti-MTLN (Proteogenix, PTX17840) and anti-GAPDH (Cell Signaling, 2118S). Bound antibodies were detected using HRP secondary antibodies (Cell Signaling, 7074P2). Primary and secondary antibodies were used at 1:1000 and 1:2000 respectively. The blots were detected using ECL (Biorad,1705060) and revealed using ChemiDoc system (Biorad). Images were produced with ImageLab (Biorad).

### Statistical analysis

Statistical significance was assessed using GraphPad Prism software. Variance within each group of data was estimated and was similar between the compared groups. *P*-values for normally distributed data were calculated using appropriate tests (two-tailed Student’s t-test or two-way ANOVA with Tukey’s adjustment). Data were expressed as mean values ± SEM. Graphical representation of differences that are statistically significant at *p* ≤ 0.05.

## Supplementary information

Table legend and supp figure legend

Supp data 1: Up-regulation of genes related to the OXPHOS pathway in efferocytic macrophages shared between different macrophage subsets in humans (monocyte-derived macrophages) and in mice.

Supp data 2: Protein expression of PERILIPN5 and MITOREGULIN in non-efferocytic (M1) or efferocytic (M1 + PMN) M1-like macrophages.-, M0-, and M2a-like macrophages.

Supp data 3: List of genes encoding proteins involved in lipolysis and found up- and down-regulated in human efferocytic M1-, M0-, and M2a-like macrophages.

Supp data 4: Lipid droplet content in non-efferocytic (M1) or efferocytic (M1 + PMN) M1-like macrophages with palmitate.

Supp data 5: Evolution of the percentage of CD11blow macrophages during zymosan-A-induced peritonitis.

Raw blot from Figure 3D

Raw blot from Figure 3D

Raw blot from Figure 5B

Raw blot from Figure 5B

## Data Availability

The RNA-seq data are available at the European Nucleotide Archive under accession number PRJEB37205.
