# Supplementary material for: HUMANIN produced by human efferocytic macrophages promotes the resolution of inflammation
Source: Cell Death Dis. 2025 Aug 28;16(1):656. doi: 10.1038/s41419-025-07909-1 (PMC12394407; doi:10.1038/s41419-025-07909-1)
Supplement: Supplementary file 1 — Table legend and supp figure legend [file 41419_2025_7909_MOESM1_ESM.docx]

**Supp data 1: Up-regulation of genes related to the OXPHOS pathway in efferocytic macrophages shared between different macrophage subsets in humans (monocyte-derived macrophages) and in mice (blood, lung and peritoneal cavity).**

**A**, Sequencing data (microarray, bulk RNA-seq or single cell [sc] RNA-seq) on macrophages experiencing efferocytosis were searched in humans and mice. The chosen datasets, along with our, are displayed on the figure according to the time of analysis after efferocytosis (6h or 24h) or after the induction of inflammation (3 days, corresponding to pro-resolving macrophages) (33, 10, 35, 34). These cells correspond to high-efferocytic macrophages. OXPHOS-related gene regulation was analyzed across these data using GSEA analysis with different databases (KEGG, REACTOME, GOBP). **B**, Enrichment scores (NES) were calculated for pathways related to OXPHOS. Significant up-regulation of OXPHOS pathway was found in both efferocytic mouse and human macrophages in these datasets.

**Supp data 2: Protein expression of PERILIPN5 and MITOREGULIN in non-efferocytic (M1) or efferocytic (M1 + PMN) M1-like macrophages.**

Immunoblots showing protein expression of PERILIPIN5 (PLIN5) and MITOREGULIN (MTLN) in human M1-like macrophages after 24 hours of efferocytosis. Human M1-like macrophages (M1) were co-incubated with human primary apoptotic neutrophils (PMN) for 24 hours, and then harvested for Western blot analysis. Results show representative blots (n=3). Stain-free control is shown as loading control.

**Supp data 3: List of genes encoding proteins involved in lipolysis and found up- and down-regulated in human efferocytic M1-, M0-, and M2a-like macrophages.** The gene list was generated based on GO database (GO:0016042 lipid catabolic process). Only genes with at least a fold change of + 1.2 and *p*-value ≤ 0.05 in one of the three types of macrophages were selected.

**Supp data 4: Lipid droplet content in non-efferocytic (M1) or efferocytic (M1 + PMN) M1-like macrophages with palmitate.**

**A**, Confocal image of lipid droplet in human M1-like macrophages after 24h efferocytosis. Macrophages with (M1 + PMN) or without incubation with apoptotic neutrophils (M1) were stimulated with palmitate (200µM) for 24 hours. This image is representative of three experiments. Lipid droplet content (Yellow) was stained using LipidTOX red while Hoechst was used to stain nuclei (blue).

**Supp data 5: Evolution of the percentage of CD11b^low^ macrophages during zymosan-A-induced peritonitis.**

HUMANIN (Hu) or vehicle (Veh.) was injected i.p. 30 min before zymosan-A (Zy., 20µg in PBS). Peritoneal lavages were performed at different time points (6h, 12h 24h and 72h) and cells were counted. **A,** Gating strategy to identify resident macrophages (a, CD11b^high^, F4/80^high^), recruited macrophages (b, CD11b^mid^, F4/80^low/mid^) and pro-resolving CD11b^low^ macrophages (c, CD11b^low^, F4/80^mid^). **B**, Flow cytometry plots of these three macrophage populations at 6h, 12h, 24h and 72h after zymosan-A injection (Zy.), with HUMANIN (Zy. +Hu) or PBS (Veh). **C**, Percentage of pro-resolving CD11b^low^ macrophages (population c) at 6h, 12h and 24h after zymosan-A injection issued from mice that received zymosan-A alone (Zy., open dark blue circles) or with HUMANIN (Zy.+Hu, open light blue circles) (n= 6-10 mice per group).
