# Supplementary figures and images for: HUMANIN produced by human efferocytic macrophages promotes the resolution of inflammation
Source: Cell Death Dis. 2025 Aug 28;16(1):656. doi: 10.1038/s41419-025-07909-1 (PMC12394407; doi:10.1038/s41419-025-07909-1)

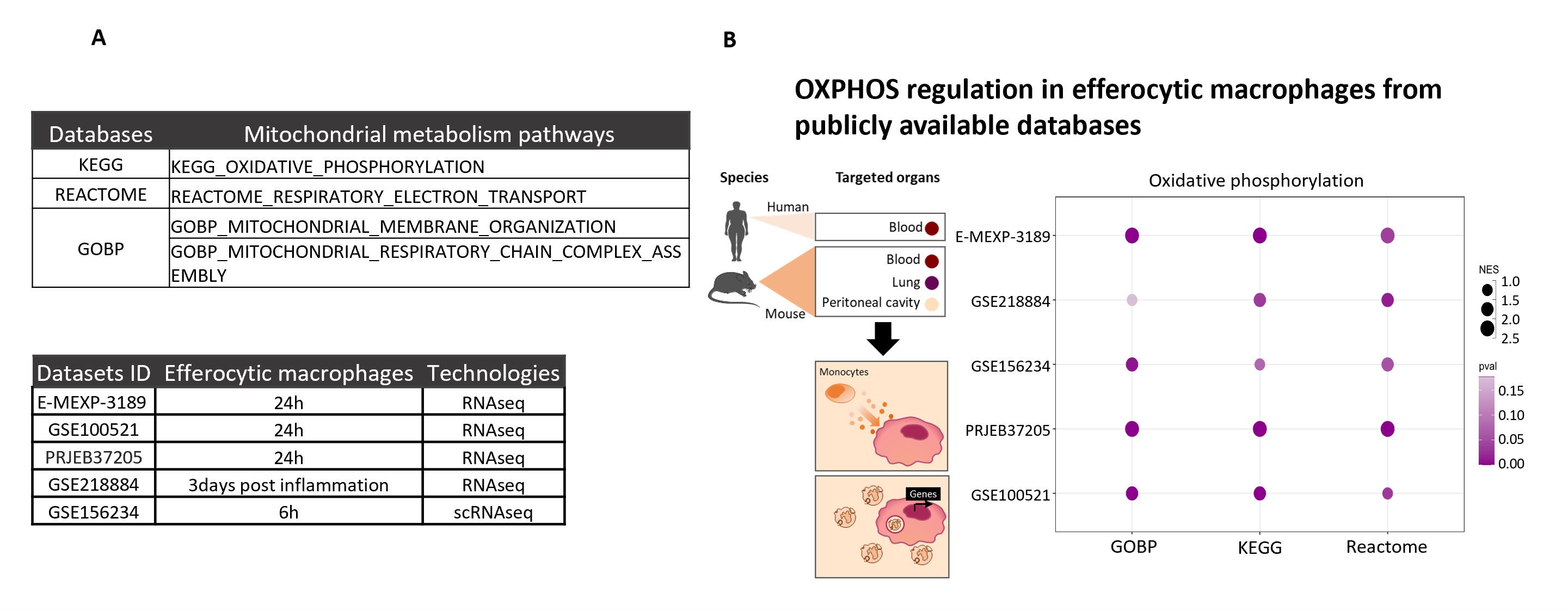

Supplement: Supplementary file 2 — Supp data 1: Up-regulation of genes related to the OXPHOS pathway in efferocytic macrophages shared between different macrophage subsets in humans (monocyte-derived macrophages) and in mice. [file 41419_2025_7909_MOESM2_ESM.png]

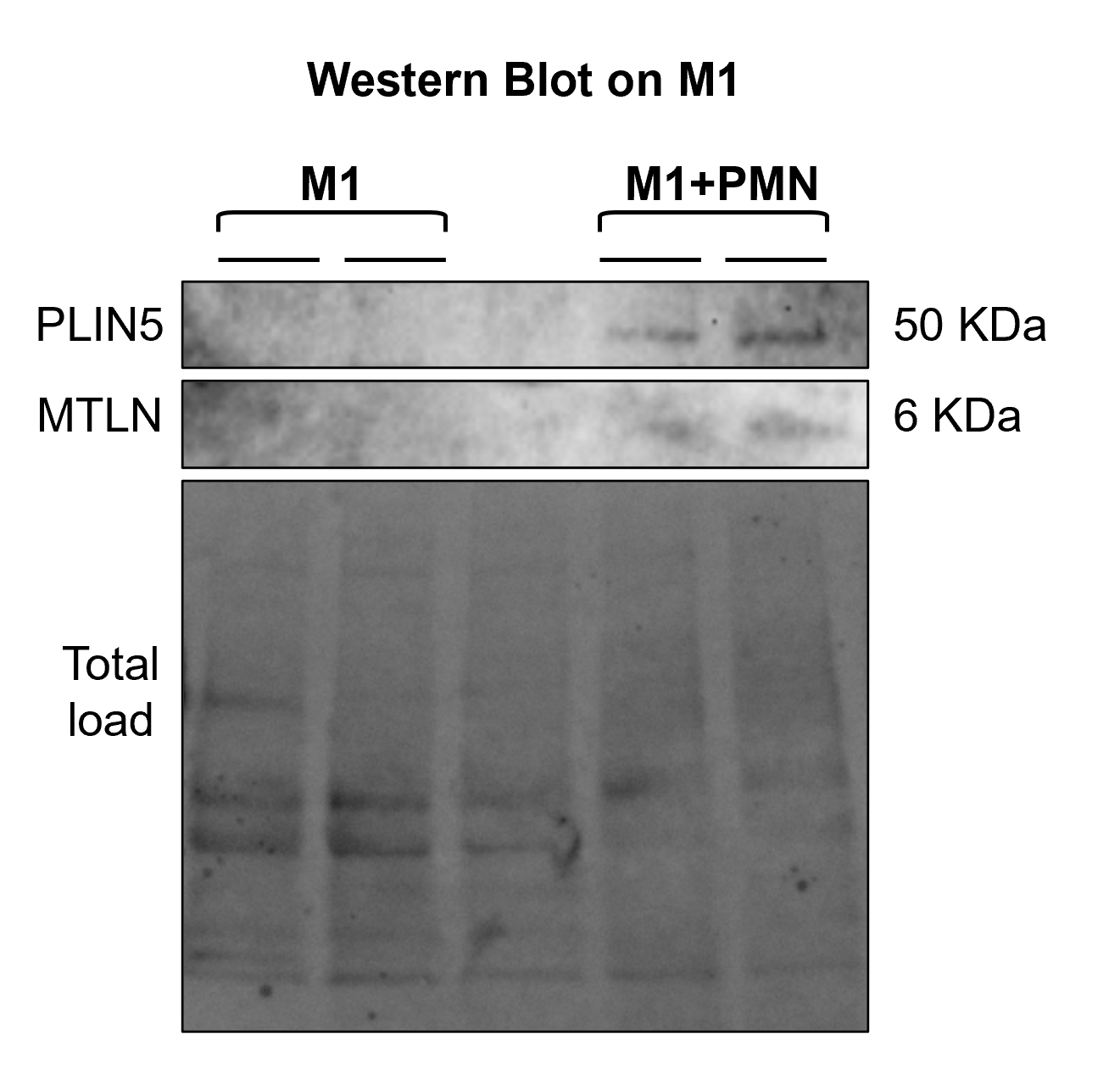

Supplement: Supplementary file 3 — Supp data 2: Protein expression of PERILIPN5 and MITOREGULIN in non-efferocytic (M1) or efferocytic (M1 + PMN) M1-like macrophages.-, M0-, and M2a-like macrophages. [file 41419_2025_7909_MOESM3_ESM.png]

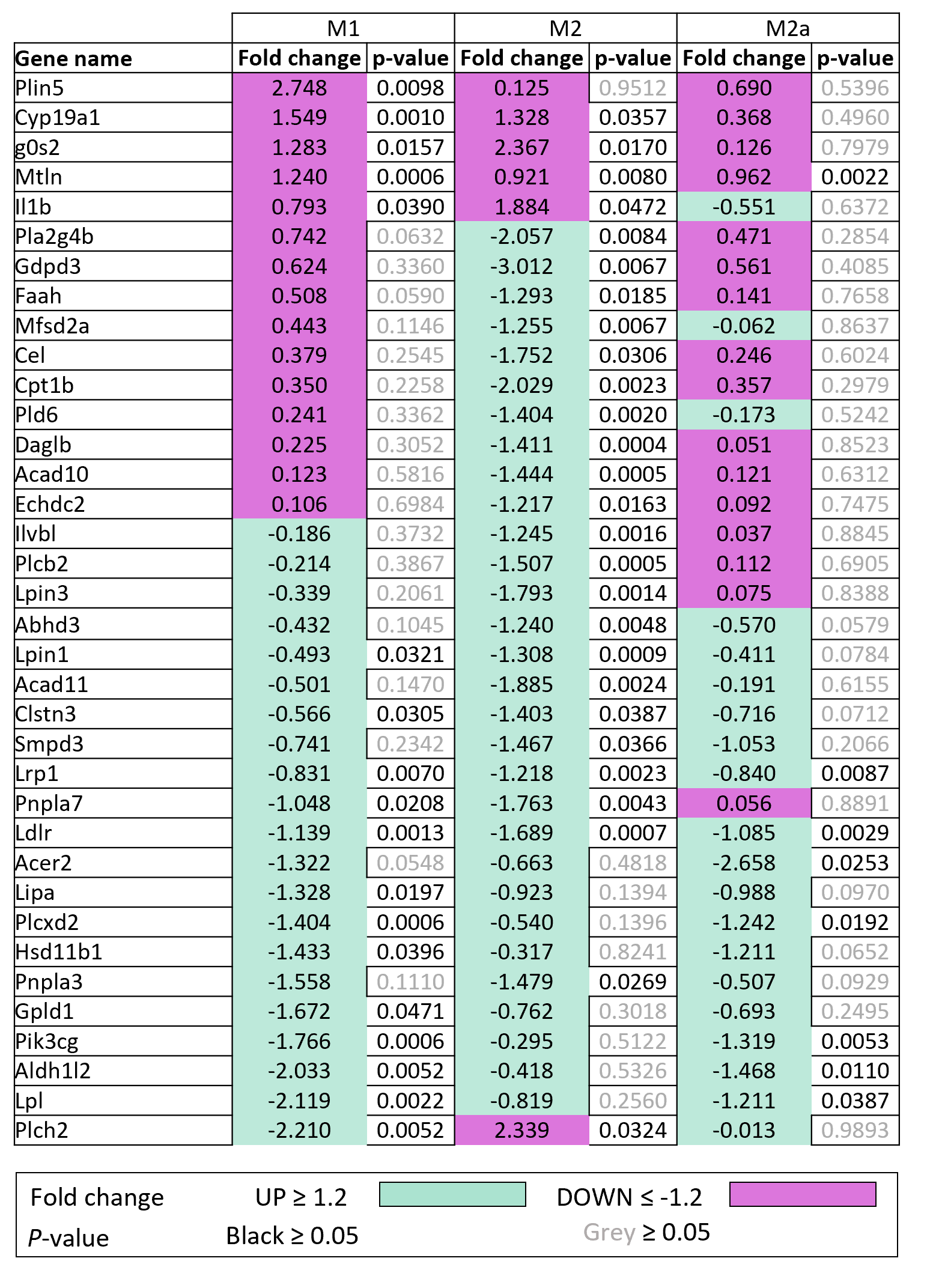

Supplement: Supplementary file 4 — Supp data 3: List of genes encoding proteins involved in lipolysis and found up- and down-regulated in human efferocytic M1-, M0-, and M2a-like macrophages. [file 41419_2025_7909_MOESM4_ESM.png]

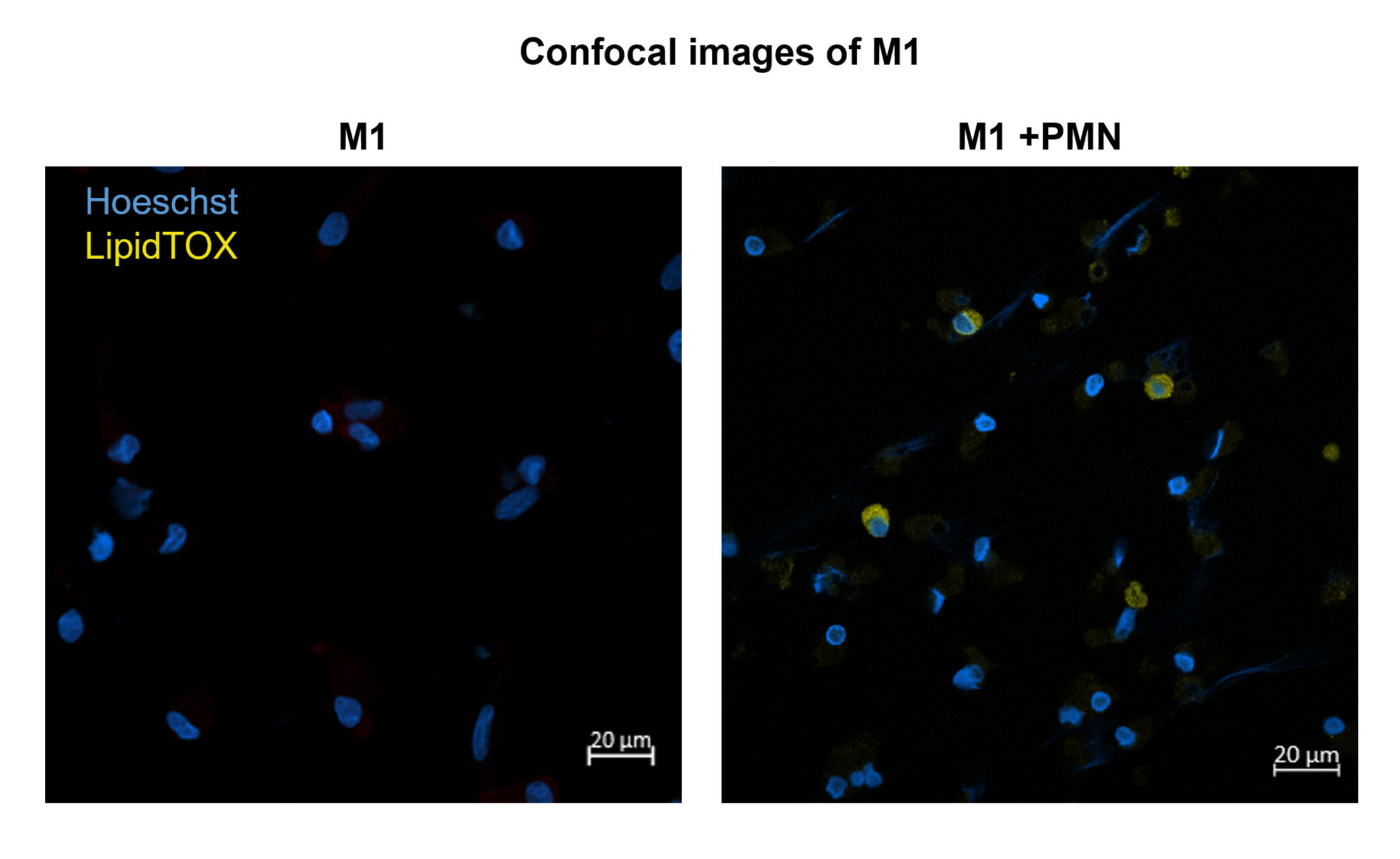

Supplement: Supplementary file 5 — Supp data 4: Lipid droplet content in non-efferocytic (M1) or efferocytic (M1 + PMN) M1-like macrophages with palmitate. [file 41419_2025_7909_MOESM5_ESM.png]

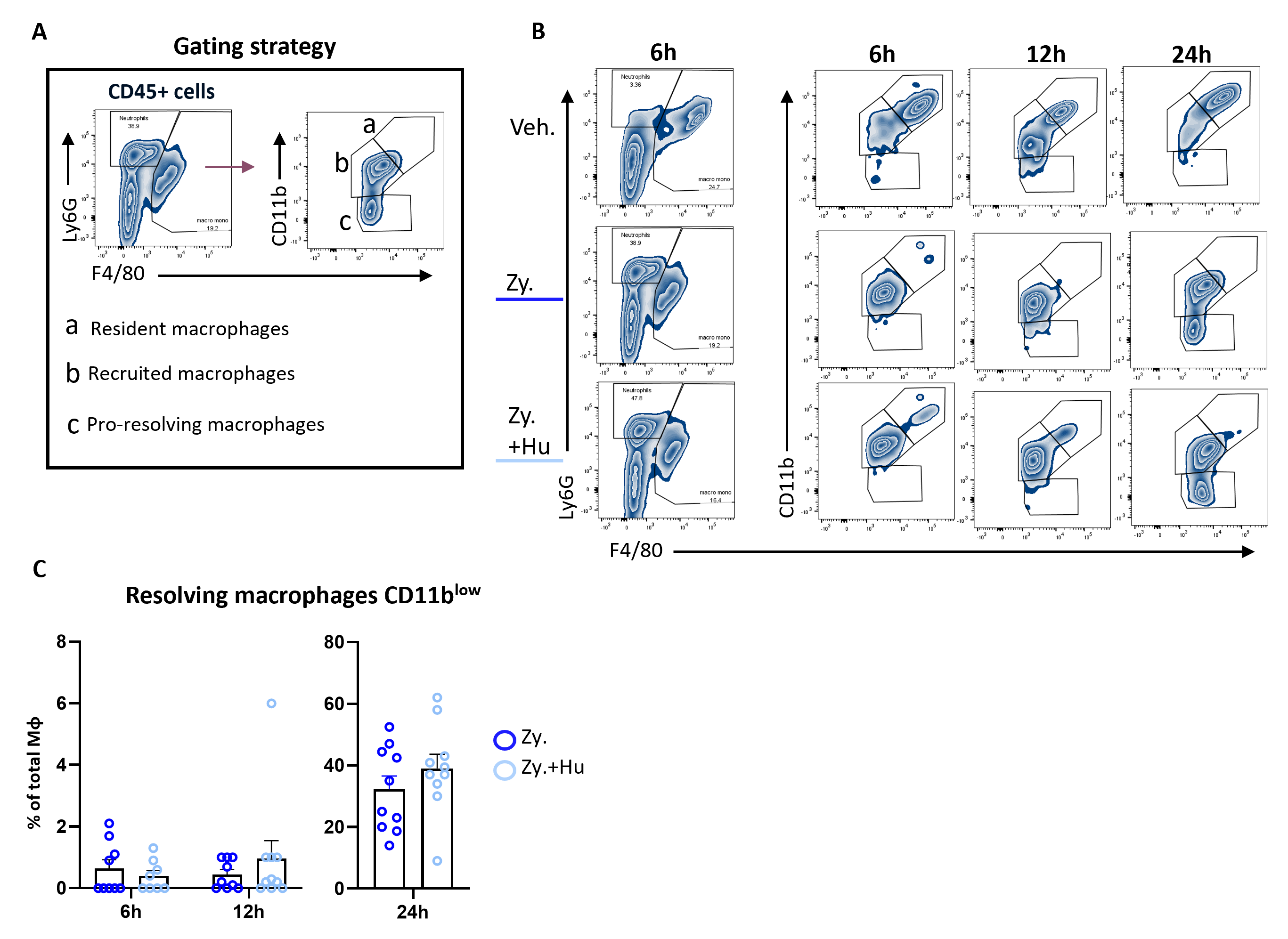

Supplement: Supplementary file 6 — Supp data 5: Evolution of the percentage of CD11blow macrophages during zymosan-A-induced peritonitis. [file 41419_2025_7909_MOESM6_ESM.png]

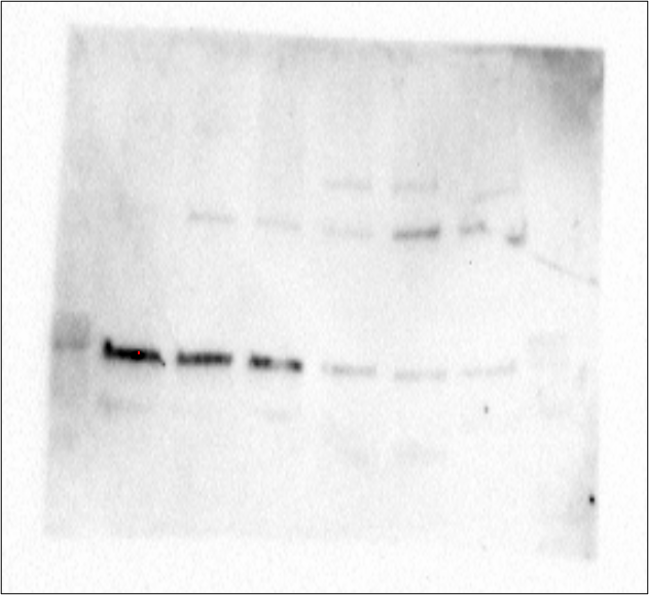

Supplement: Supplementary file 7 — Raw blot from Figure 3D [file 41419_2025_7909_MOESM7_ESM.png]

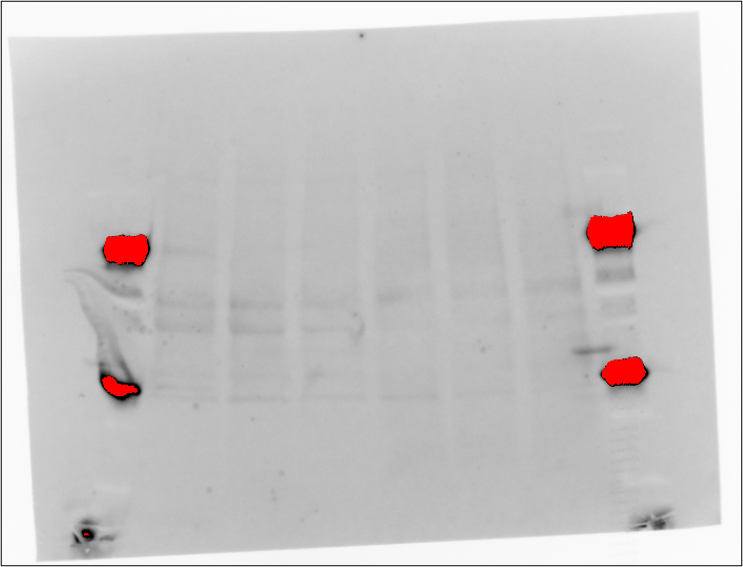

Supplement: Supplementary file 8 — Raw blot from Figure 3D [file 41419_2025_7909_MOESM8_ESM.png]

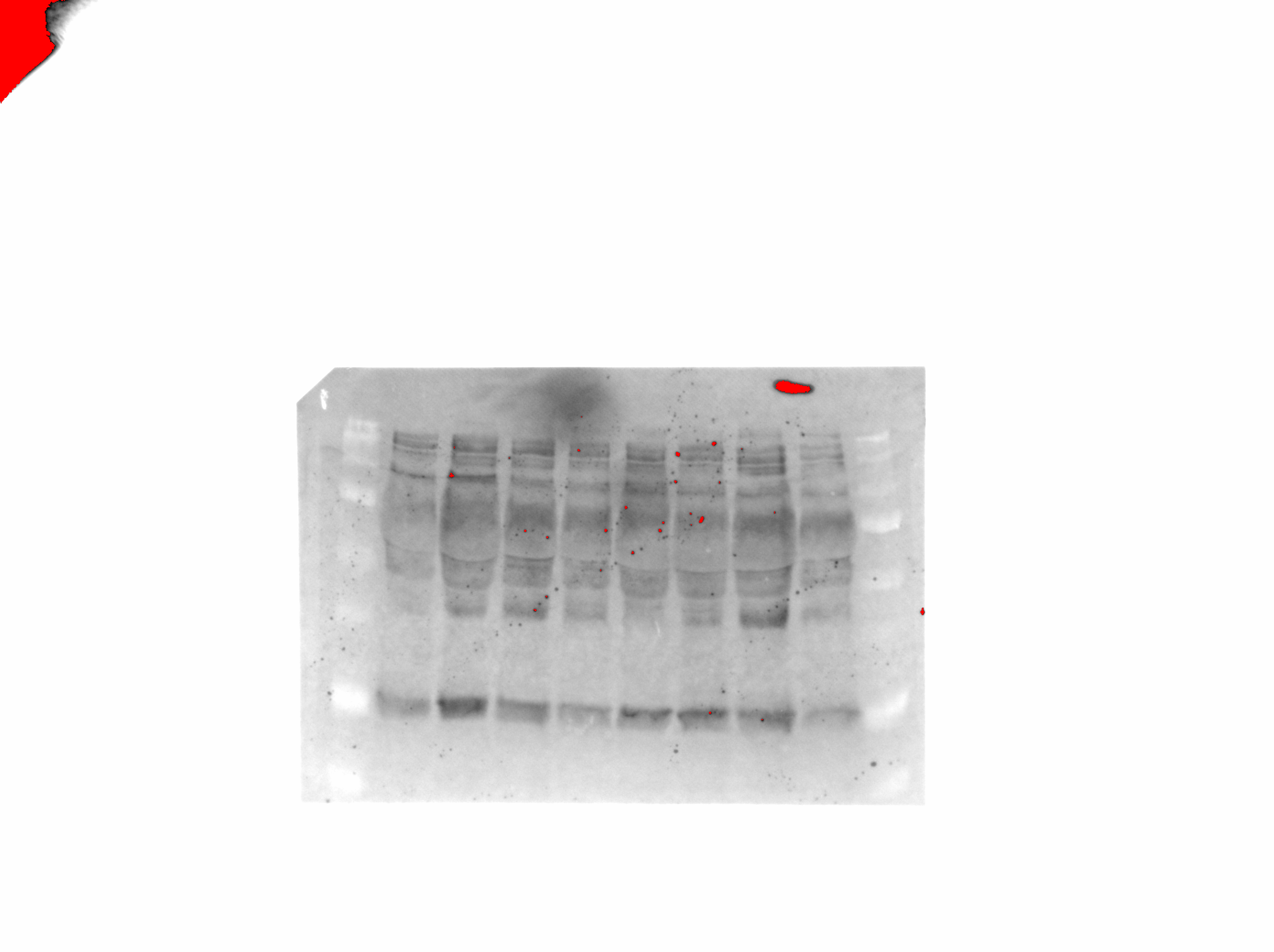

Supplement: Supplementary file 9 — Raw blot from Figure 5B [file 41419_2025_7909_MOESM9_ESM.png]

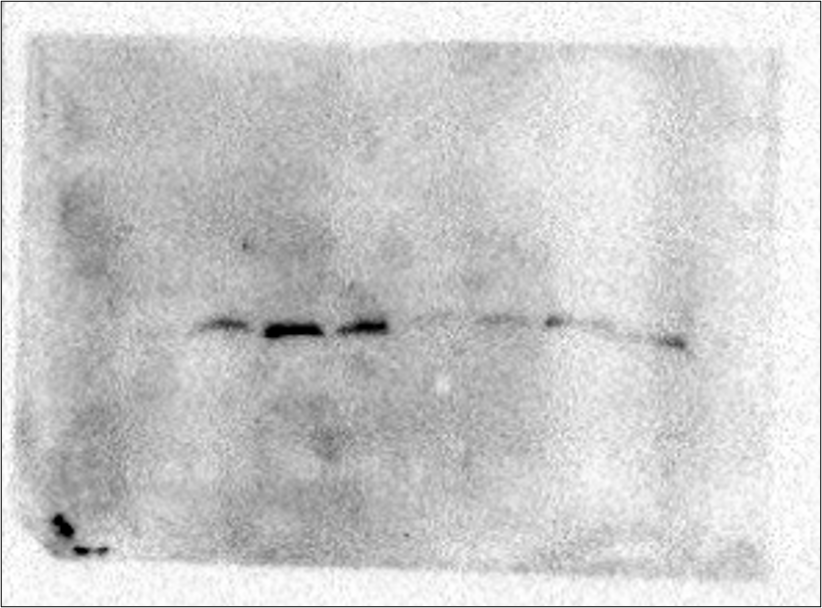

Supplement: Supplementary file 10 — Raw blot from Figure 5B [file 41419_2025_7909_MOESM10_ESM.png]
